# A novel method for rapid and sensitive metagenomic activity screening

**DOI:** 10.1016/j.mex.2018.06.011

**Published:** 2018-06-20

**Authors:** Meiling Shang, Victor J. Chan, Dominic W.S. Wong, Hans Liao

**Affiliations:** a Western Regional Research Center, USDA-ARS, Albany, CA, USA; b Cargill Biotechnology Development Center, Minneapolis, MN, USA

**Keywords:** Metagenomic library, Functional screening, Activity screening, Split-pool sub-library screening, Substrate gel microtiter assay plate

## Abstract

Direct cloning of metagenomes has proven to be a powerful tool for the exploration of the diverse sequence space of a microbial community leading to gene discovery and biocatalyst development. The key to such approach is the development of rapid, sensitive, and reliable functional screening of libraries. The majority of library screen have relied on the use of agar plates in petri dishes incorporating the target enzyme substrate for activity detection of positive clones (Iqbal et al. [1], Knietsch et al. [2], Popovic et al. [3]). In this article, a novel method is described consisting of: (1) formulation and application of substrate gel microtiter assay plates, (2) screening of libraries of clones in split pools in the wells of the assay plate, and (3) progressive enrichment and isolation of individual positive clones. The method has been successfully used in the rapid discovery of novel genes and enzymes from rumen microbial metagenome with high efficacy.

• Novel substrate gel assay plates for activity screening with localized and intensified signals.
• Rapid and complete screening of library clones in split pools.
• Progressive enrichment scheme as a refining step for isolating target gene.

## Graphical abstract

**Figure:**
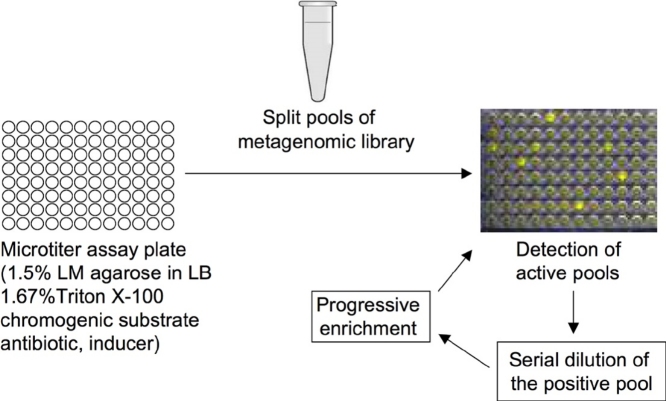

## Method details

### Background

Microbial diversity provides a vast genetic resource for the discovery of new genes, enzymes, natural products, and bioactives with impacts on industrial and biotechnological applications. However, only less than 1% of the microorganisms in the natural environment can be cultured in traditional laboratory conditions. For the last two decades, metagenomic research has demonstrated that direct cloning of collective genomic DNA provides a powerful tool for exploring the diverse sequence space of uncultured microbes [4]. The general methodology involves sampling of environmental microbes, direct extraction of the metagenome, restriction of the total DNA, cloning of the DNA fragments into a suitable vector, and transformation of the recombinant DNA into a host for construction of metagenomic libraries [5]. This is followed by a key step of sequence-based or function-based screening of the library [[5], [6], [7]]. The development of next-generation sequencing technology and advanced bioinformatics has significantly improved the efficiency of sequence-based screening. However, sequence-based search is limited to the screening for homologs of already known sequence motifs, and quite often obtaining incomplete gene sequences. In contrast, function-based screening involves cloning and functional expression of gene-containing fragments in a heterologous host, and allows the detection of active clones, which is particularly desirable for identifying novel biocatalysts [8]. The enzyme activity and biochemical parameters can often be revealed during the screening. One challenge of functional metagenomic is the development of sensitive, reliable and rapid assays for screening libraries. This report describes a novel method for rapid and sensitive screening and isolation of genes/enzymes of interest from complex metagenomes, with the following unique features: (1) the formulation and application of substrate gel microtiter assay plates for rapid screening of library of clones in split pools, and (2) a progressive enrichment and isolation of individual positive clone and the target gene.

### Preparation of substrate gel microtiter assay plate

In the general scheme, a metagenomic library was split into small pools (sub-libraries) for culturing in 96-well microplates (culture plates). The sub-libraries grown overnight in the culture plate were then transferred to premade substrate gel microtiter assay plates. The substrate gel was formulated to enable the localization and confinement of the reaction products, resulting in intensified signal for detection [9].

The following is a typical protocol for preparing substrate gel microtiter assay plates. In one test tube, low-melt agarose of 1.5% (w/v) was melted into Luria Bertani (LB) or other suitable growth medium and equilibrated to 50 °C. In a separate tube, buffer, detergent, antibiotic, inducer, and substrate were mixed in the growth medium and equilibrated to 50 °C A typical example would include 50 mM buffer, pH 6.5, 1.67% (v/v) detergent (Triton X-100), 50 μg/ml antibiotic, 2 mM expression inducer, and 2 mM chromogenic substrate added to LB medium to a final volume of 25 ml and equilibrated to 50 °C. The two tubes were combined, mixed and poured in 10 ml aliquots into a reservoir (for multichannel pipettors). Using an 8-channel pipettor fitted with 1.2 ml tips, the combined mixture was quickly dispensed at 50 μl per well to 96-well microtiter plates (half area, flat bottom, polystyrene). A volume of 50 ml would be sufficient for preparing 9 substrate gel microtiter assay plates. After the gel was set, the assay plates were used immediately, or could be stored at 4 °C for several weeks. The substrate gel mix should contain three basic components: (1) a buffer at a suitable pH (dependent on the pH optimum of the enzyme activity to be detected), (2) a detergent Triton X-100, (3) a suitable substrate for detecting expression of the target gene. The type of substrate could be chromogenic, flourogenic, or soluble/insoluble dye-crosslinked, enabling the enzyme reaction product to be quickly visualized and/or measured by a plate reader. The inclusion of antibiotics and inducers would depend on the type of libraries, which can be constructed based on *Escherichia coli*, bacteriophage, phagemid, and so on.

### Application of substrate gel microtiter assay plates for screening metagenomic libraries

In a general scheme, 5 μl of an overnight culture of each sub-library (see below) in the culture plate was transferred into individual wells of a premade microtiter assay plate. The assay plate was sealed with permeable membranes, incubated, and monitored for the formation of reaction products due to expression of the target gene. Wells showing product formation consisting of active pools were enriched to isolate individual positive clones. The following protocol example describes the use of substrate gel microtiter assay plates for screening feruloyl esterase genes.

Substrate gel microtiter assay plate #1: The assay plate was prepared for screening feruloyl esterase phagemid library as follows. In one tube, low-melt agarose of 1.5% (w/v) was melted into LB medium and cooled to 50 °C. In a separate tube, 50 mM sodium phosphate buffer, pH 6.5, 1.67% (v/v) Triton X-100, 50 μg/ml ampicillin, 2 mM IPTG and 70 mM *p*-nitrophenyl ferulate (enzyme substrate) in DMSO were added to LB medium and equilibrated to 50 °C. The two tubes were combined, mixed, and dispensed to microplates in 50 μl aliquots per well and allowed to set at room temperature.

Substrate gel microtiter assay plate #2: An alternative assay plate used the synthetic substrate ethyl ferulate. The substrate was dissolved in DMF and added to the LB agar to a concentration of 0.15% (w/v) prior to autoclaving. After cooling to 50 °C, antibiotic was added and the medium mix was poured into microplates as described above. The substrate ethyl ferulate caused the medium to become opaque once it was set.

An aliquot of a phagemid library (constructed from rumen microbial metagenome) containing 1 × 10^5^ cfu was diluted in 80 ml of LB with ampicillin (50 μg/ml). A volume of 0.1 ml of the diluted phagemid was dispensed per well into eight 96-well microplates. Each well therefore contained about 125 cfu per well. Each individual well represented a sub-library (sub-populations) of the original library. The plates were sealed with permeable membranes and incubated overnight at 850 rpm and 37 °C in a microplate incubator shaker. These culture plates were then used to inoculate premade substrate gel microtiter assay plates, and afterwards stored at 4 °C as the master plate containing sub-libraries.

**Fig. 1:**
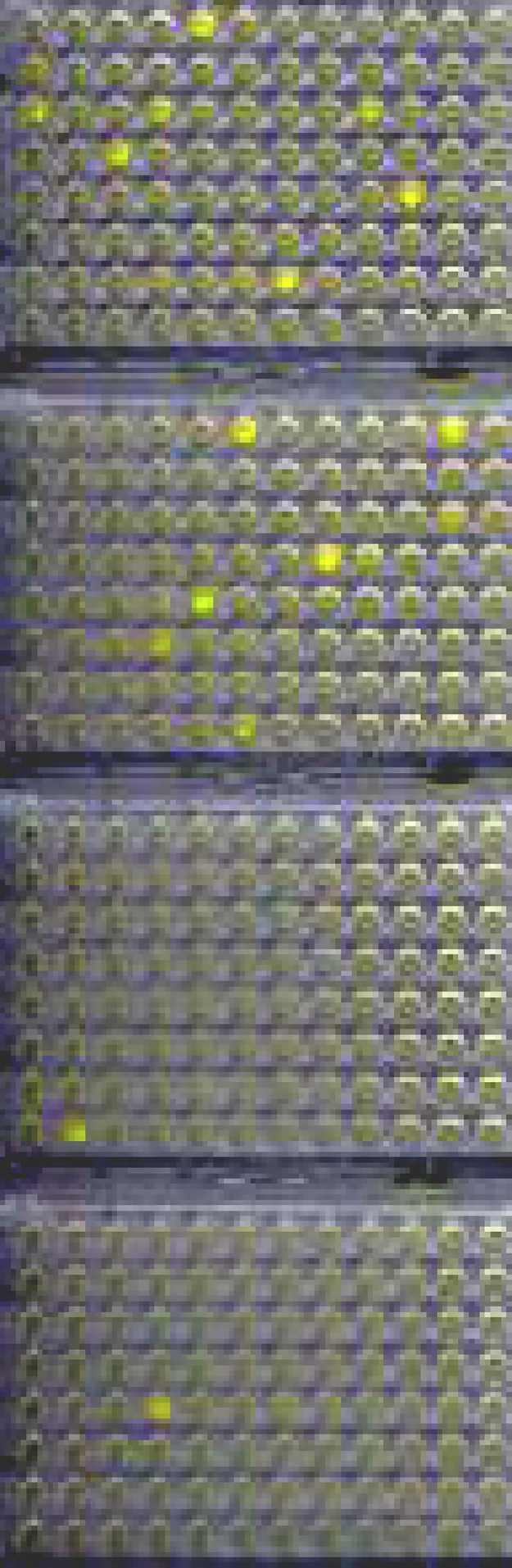
Detection of chromogenic product, *p*-nitrophenol indicating active pools in a substrate gel assay plate. Each active pool contained a positive clone expressing feruloyl esterase activity, resulting in the conversion of the substrate *p*-nitrophenyl ferulate to the colored product.

The overnight cultures in individual wells of the culture plates were transferred in 5 μl volume onto the wells of premade assay plates. The assay plate was sealed with permeable membrane and incubated without shaking at 30 °C or 37 °C overnight. Typically, with *p*-nitrophenol ferulate as substrate, the wells containing active pools (that is, containing a positive clone in the cell population) would produce the yellowish reaction product, *p*-nitrophenol, detectable within 8 h at 37 °C by a plate reader (Fig. 1). For ethyl ferulate, clearance of the gel (halo formation against an opaque background) in a well indicates active pools. Wells showing FAE activities were subjected to the enrichment step as described below.

### Progressive enrichment for isolation of an active clone from a positive pool

A serial dilution scheme was developed to enable quick enrichment and isolation of the active clone(s) from the population in the positive pool. Each active pool identified above was serially diluted in a 96-well microplate, with each column representing one complete round of serial dilution, repeating 12 times across the plate. A serial dilution started from row A continuing to row H (8 dilutions). In a detailed scheme, an aliquot of 2.5 μl of the positive sub-library (from the master plate containing sub-libraries) was added to 122.5 μl of growth medium in row A, resulting in a 1:50 dilution. Next, 25 μl from row A was diluted in 100 μl growth medium in row B, resulting in a further 1:5 dilution. This dilution step was continued to row H yielding a final dilution of 8 × 10^5^ in row H. The plate was sealed with a permeable membrane and incubated overnight in a microplate incubator shaker (850 rpm, 37 °C). The overnight culture (5 μl) in each well was transferred to and assayed on premade substrate gel assay plates as described.

The most dilute well of the serial dilution plate yielding positive detection was selected for another round of serial dilution or for a final step of isolation of individual active colonies as follows. A small volume of the culture in the positive dilut well was titered to 10^−6^ cfu, and spread on several LB ampicillin agar plates. The overnight colonies were individually picked to inoculate into 96-well microplates filled with LB ampicillin (culture plates), and incubated in a microplate incubator shaker (850 rpm, 37 °C, overnight). A 5 μl aliquot of the overnight culture in each well was assayed on substrate gel assay plates. Typically, 200–400 individual colonies were picked for assay and 1–5% of wells were positive. A Q-Pix colony picker could be used to quickly array colonies in microplate well. Positive clones were then cultured for sequencing.

## Unique features and advantages

The methodology for functional screening of metagenomic libraries was developed with the following unique features. (1) The substrate gel microtiter assay plate was particularly designed for the high efficiency screening. Chromogenic and fluorogenic substrates frequently used for enzyme activity assays have been found unsatisfactory for library screening in our initial work if the substrate agar was prepared in petri dishes or omni plates as commonly used in some protocols [1, 2, 3] . The minute quantity of the enzyme reaction product combined with its rapid diffusion through the agar medium rendered the screening unreliable in capturing the signal. Compartmentation of the substrate gel in microplate wells had the effect to contain and minimize signal diffusion, significantly increased the sensitivity of the assay, and allowed rapid measurement of the activity and unambiguous identification of positive clones even at very low activity level. (2) The incorporation of a detergent at sub-cytotoxic concentrations was to enhance the diffusion of substrate/enzyme across the cell membrane, and enzyme action on the substrate. The type of detergent and its concentration were used for optimum formulation to maximize permeability and minimize toxicity. A gel matrix platform containing both growth nutrients and enzyme substrates would then allow for simultaneous maintenance/growth of the clones and activity monitoring of gene expression. (3) The enrichment strategy contained two key steps: a stepwise reduction of the complexity of pool, and repeated dilution and sampling of the positive pool. This strategy enabled rapid isolation of individual positive clones in the sub-library pool. The serial dilution approach was derived in full extension of the efficacy of the substrate gel assay being able to detect the activity of a very few active clones.

## Additional information

In the present example, screening by both types of FAE assay plates could be performed for extended periods of time with continuing signal accumulation up to 20 h. The two screenings were conducted in parallel to confirm the activity and reduce false positives. A total of seven FAE genes (numbered C1–C7) have been isolated from rumen microbial metagenomic library.

**Fig. 2:**
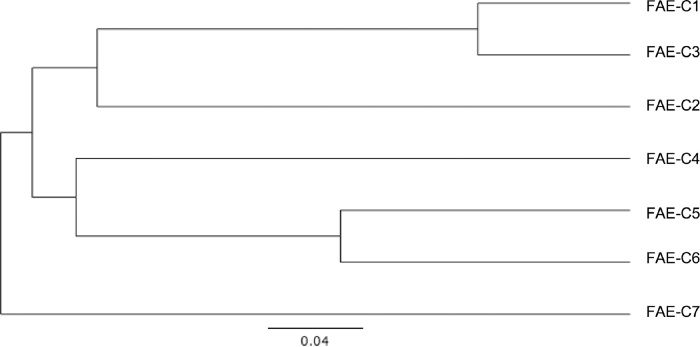
Phylogenetic tree of FAE sequences. Genetic distance model: Tamura-Nei. Tree build method: Neighbor-joining. Resample by: bootstrapping.

A BLAST of the sequences shows highest bit score in a range of 32 to 48% identity. In the case of C1, C2, and C3, the top hit scores refer to hydrolases of α/β family. The FAE-C4 shows 41% identical to the fungal *Orpinomyces* sp. PC-2 FAE (Table 1). The rest of the clones have hit scores referring to esterases of some type. All taken together, these seven FAE genes are diverse and novel. Phylogenetic tree analysis suggests that the FAE sequences fall within two major clades (Fig. 2). C1 and C3 have a common node, so do C5 and C6, with the number of support for both nodes equal to 100 percent. This relationship is also reflected in the sequence homology. For example, the C5 and C6 sequences share 84% identity and 90% similarity. In terms of calculated distance values, C7 forms the outgroup showing the most divergent from the other sequences. C1, C3, C5 and C6 have less divergence, while C4 and C7 are intermediates. All these results provide further support on the diverse and novel nature of the genes.

**Table 1:** FAE genes and proteins.

Clone Name	Reference Sequence	% Identity to Ref Seq
FAE-C1	ZP_01061020.1 Hydrolase of α/β Family	47
FAE-C2	ZP_01061020.1 Hydrolase of α/β Family	45
FAE-C3	ZP_01061020.1 Hydrolase of α/β Family	46
FAE-C4	AAF70241 Feruloyl Esterase A	41
FAE-C5	ZP_01122099.1 probable lipase/esterase	32
FAE-C6	YP_169760.1 hypothetical protein lipase/esterase	34
FAE-C7	ZP_01777732 putative esterase	48

The methodology described in this report has been successfully applied in the discovery of arabinanase, xyloglucanase, bifunctional mannanase-glucanase, acetylxylan esterase, and a number of others, totaling 38 novel genes [[10], [11], [12], [13], [14], [15]]. The method should be applicable as a universal tool for screening metagenomic libraries with high efficacy.

## Conclusions

The methodology described in this report consists of approaches radically departed from conventional methods used for screening metagenomic libraries. It has the unique advantages of: (1) screening library of clones in split pools; (2) localizing and intensifying positive signals on substrate gel assay plates; and (3) progressive enrichment and isolation of positive clones. The method significantly enhances the success rate in gene discovery. The method has been developed and used successfully in our various projects to discover numerous hard-to-find novel genes/enzymes within a short time period.
